# Pain management in trauma: A review study

**DOI:** 10.5249/jivr.v8i2.707

**Published:** 2016-07

**Authors:** Alireza Ahmadi, Shahrzad Bazargan-Hejazi, Zahra Heidari Zadie, Pramote Euasobhon, Penkae Ketumarn, Ali Karbasfrushan, Javad Amini-Saman, Reza Mohammadi

**Affiliations:** ^*a*^Department of Anesthesiology, Critical Care and Pain Management, Imam Reza Hospital, Kermanshah University of Medical Sciences, Kermanshah, Iran.; ^*b*^Karolinska Institutet, Stockholm, Sweden.; ^*c*^Department of Psychology, College of Medicine at Charles R. Drew University of Medicine, and Science & David Geffen School of Medicine at UCLA, Los Angeles, CA, USA.; ^*d*^Department of Anesthesiology and Pain Management, Mahidol University, Bangkok, Thailand.

## Abstract

**Background:**

Pain in trauma has a role similar to the double-edged sword. On the one hand, pain is a good indicator to determine the severity and type of injury. On the other hand, pain can induce sever complications and it may lead to further deterioration of the patient. Therefore, knowing how to manage pain in trauma patients is an important part of systemic approach in trauma. The aim of this manuscript is to provide information about pain management in trauma in the Emergency Room settings.

**Methods:**

In this review we searched among electronic and manual documents covering a 15-yr period between 2000 and 2016. Our electronic search included Pub Med, Google scholar, Web of Science, and Cochrane databases. We looked for articles in English and in peer-reviewed journals using the following keywords: acute pain management, trauma, emergency room and injury.

**Results:**

More than 3200 documents were identified. After screening based on the study inclusion criteria, 560 studies that had direct linkage to the study aim were considered for evaluation based World Health Organization (WHO) pain ladder chart.

**Conclusions:**

To provide adequate pain management in trauma patients require: adequate assessment of age-specific pharmacologic pain management; identification of adequate analgesic to relieve moderate to severe pain; cognizance of serious adverse effects of pain medications and weighting medications against their benefits, and regularly reassessing patients and reevaluating their pain management regimen. Patient-centered trauma care will also require having knowledge of barriers to pain management and discussing them with the patient and his/her family to identify solutions.

## Introduction

According to World Health Organization (WHO) injury is the leading cause of death among men and women age 15 to 44 years and will be the third leading cause of death and disability in all ages in 2020.^1^ Road traffic crash (RTC) is one of the main causes of injury responsible for approximately 50 million injuries, per year, worldwide. ^2^

Complaint of pain is one of the most prevalent condition among trauma patients in the emergency room settings. Pain management of the elderly and children is especially challenging because these patients often present with multiple chronic medical conditions or heightened anxiety, respectively.^3^ Trauma patients, also, report low satisfaction with their pain management. ^4^ In addition, management of trauma patients has been one of the most resource-intensive medical care performed in resource challenged emergency room settings.^5,6^

Trauma patients include a wide spectrum of physiologically various patient populations including healthy young athletes, vulnerable children, and frail elderly. To provide optimum pain management care to these patients, it is necessary that practitioners are well prepared to and current with utilizing modern evidence-based knowledge and practices. Additionally, trauma patients who present with multiple injuries, substance abuse, delayed care, as well as psychological and emotional issues complicate the care process.^7,8^

Providing the appropriate and timely pain management to trauma patients is not only the patient right, also it prompts early healing, reduces patient’s Stress Response (SR), shorten hospital length of stay, lowers costs, diminishes risk of chronic pain due to neuroplasticity, and ultimately reduces rate of morbidity and mortality.^9-13^ Physicians often report concerns about increasing the pain medication prescription dose or analgesia for pain management. This roots in their worries about patients' adverse physiologic reactions such as risk of addiction, instability in hemodynamic situation and depression of respiratory system. Others have pointed to lack of standardized protocols for analgesia usage for management of acute pain.^7,10,14-16^

The specific aim of this review study is: 1) to describe pain treatment and management modalities in trauma injury, emphasizing on pharmacologic interventions, invasive and noninvasive pain management techniques and; 2) to introduce selective approaches based on the carefully assessment of nature and extent of injuries provides optimum analgesia with a minimum of adverse effects.

## Methods

**Inclusion Criteria**

Our literature review included electronic and manual search to identify all relevant publications. The electronic search was based on the published studies in English, which were identified on Pub Med, Google scholar, Web of Science, and Cochrane database between 2000 and 2016. We used combination of key terms such as acute paint management, trauma, emergency room and injury. All types of studies were included in this review such as randomized control trials (RCTs) or descriptive and analytic studies (cohort or case-control), review articles, guidelines and protocol. We excluded case reports, case series, clinical audits and non-English publications.

**Search Strategy and Study Selection Criteria **

The 1st and 2nd authors, independently, screened all the published studies that were identified during their initial database search for inclusion criteria based on the information provided on the titles, abstracts, and MeSH terms. Studies were included if their titles contained one or more terms related to pain management, trauma or Emergency Room (ER) care, and injury. If it was not clear whether the study met inclusion criteria, the study team reviewed the full paper. They also reviewed selected bibliographies for additional papers. Studies were excluded if their sample comprised mostly or entirely of non-trauma patients, pain management was not the aim of the study, or procedural sedation and analgesia were the main focus of the study. Opinion articles also were excluded. All the discrepancies between reviewers were discussed and resolved, subsequently.

Our electronic and manual searches covered the period between 2000 and 2016. More than 3200 studies were identified. After initial review, 560 studies that met the study inclusion criteria were selected for review. 

## Results

**Recommended order of actions in trauma pain management:**

**1.Assess pain as part of the general patient care: **

In the event of acute pain, it is recommended to carefully assess patient based on OPQRST. OPQRST^17^ stands for Onset of the event, Provocation or palliation, Quality of the pain, Region and radiation, Severity, and Time (history). In addition to documentation of OPQRST, the patients’ subjective assessment of their pain should be recorded in their medical chart (SOAP note) since there are misconceptions and culturally determined beliefs about pain. It must be noted, for example, sometime patients tolerate pain as a sign of "manhood". Furthermore, patients’ knowledge and attitude towards behavioral pain control techniques may be a determining factor for successful pain management. With the same token successful pain management partly dependents on the positive relationship between the patients and their caregivers/families. ^18^ The aforementioned factors are important while taking history at the time of admission and assessing patient’s pain using Visual Analogue Scale (VAS) and Verbal Rating Scale (VRS). The careful examination and understanding of the complexity and multifactorial nature of pain ultimately determine the rational for prescribing analgesic medications and recommending a pain management plan. 

**Use age-appropriate pain assessment scale as following: **

Assessment of pain in traumatic patients may be difficult because of patient’s lack of consciousness or other impediments such as head trauma, facial injury or alcohol/ drug abuse. Also, many trauma patients experience high level of sudden emotional trauma, fear, anxiety and psychosomatic distress, which make it difficult for the physician to make reliable assessment and relevant interpretation. In addition, the size of the wound or the amount of blood loss does not correlate well with extent of tissue injuries, intensity of pain, or analgesic requirements, making accurate assessment of the pain problematic. However, a number of standardized unidimensional pain scales have been developed for acute pain assessment. Some of the most popular assessment tools based on the patient’s age are as follow:

Age<4 yrs: Consider using an observational scale such as

o Faces, Arms, Legs, Cry, Consolability (FALCC). Each domain is scored from 0-2, which results in a total score between 0-10. Zero indicating relaxed and comfortable; 1-3 = Mild discomfort; 4-6 = Moderate pain, and 7-10 = Severe discomfort/pain

o Children’s Hospital of Eastern Ontario Pain Scale (CHEOPS). It includes six categories (cry, facial, verbal, torso, touch, legs), each with 3-4 levels of care, with the total score= 4-13. 

Age 4-12 yrs: Consider using a self-report scale such as 

o Faces Pain Scale (FPS) is an illustrative scale of drawings of face expressions that is useful in children and persons who has language barriers. Children point to face that represents their pain using scores 0-6 (happy face to sad face)

o FPS-revised, using scores 0-10. 

o Wong-Baker Faces, using scores 0-10 (no pain to hurt worst)

Age >12 yrs: Consider using a self-report scale such as 

o Numerical Rating Scale (NRS). In this scale pain rates from 0 to10 (no pain to worst possible pain).

o Visual Analogue Scale (VAS). Patients marks the severity of pain on line.

o Verbal Rating Scale (VRS). The patient rates the pain on a Likert scale verbally, e.g. "none", "mild pain", "moderate pain", "severe pain", "very severe pain" or "worst possible pain".

**2. Pharmacological/ non-pharmacological intervention: **

Pain management in trauma is classified under acute pain management (Table 1). Frequently, in the ER most efforts are made to treat the pathology. It is important to pay attention to both symptoms (including pain) and pathology (e.g. fracture).^19-21^

**Table1:**
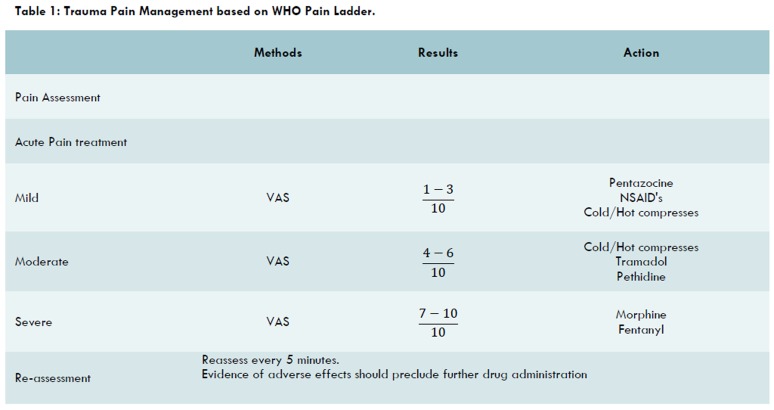
Trauma Pain Management based on WHO Pain Ladder.

**Multimodal Techniques for Trauma Pain Management:**

Administration of rapidly acting intravenous agents in small doses at frequent intervals until pain relief is achieved is recommended. This allows the practitioner to determine the patient's basal requirements before starting long-acting medications or patient-controlled analgesia. Hypotension in response to analgesics is the most commonly indicative of hypovolemia and should prompt a search for occult hemorrhage while further resuscitation occurs. While the total elimination of pain is not practically attainable, multimodal pain management techniques including the administration of two or more drugs with different alleviating mechanisms can provide suitable analgesia. These drugs may be administered via the same route or by different routes.^22,23^

Systemic pharmacological techniques are the mainstay of therapy during the emergency phase of a trauma case. Intravenous titration of small doses of opioid allows adjustment for the individual variations. Intramuscular or subcutaneous injection of opioids generally is not as effective and will be inadequate in the presence of hypovolemia. Long-acting opioids such as ms-contin (morphine sulfate controlled-release) drug and Kapanol usually are not recommended in this phase.

The current practices for trauma pain management include but are not limited to the following single modalities: (1) central regional (i.e., neuraxial) opioid analgesia; (2) Patient-Controlled Analgesia (PCA) with systemic opioids; and (3) peripheral regional analgesic procedures, including but not limited to intercostal blocks, interpleural catheter, plexus blocks, and local anesthetic infiltration of incisions.

The selection of analgesic should be based on the main assessment score and the WHO Pain Ladder. The WHO Pain Ladder was developed in 1986 as a conceptual model to guide the management of cancer pain.^24^ There is now a worldwide consensus promoting its use for the medical management of all pain associated with serious illness, including pain from wounds. 

Trauma patients often have severe pain with score equal to 7-10/10. If severe pain persists or increases, strong opioids are indicated intravenously (Table 1 and Table 2).

**Table 2:** Pharmacological and equianalgesic characteristics of some common opioids.

Opioids	Relative potency to morphine P.O.	Main Receptor activity	Routes of administration	O: P ratio	Onset (min)	Peak (min)	T 1/2 (h)	Duration of pain relief (h)
Fentanyl	150	µ agonist	IV, ED. Transmucosal, Transdermal	-	5 IV/TM	-	2 IV	0.4- 0.5 IV 72 h TD
Phenazocine	5.0	µ agonist	PO, PR	1:0.4	20	45-60	?	6
Methadone	1.0 single 3-4 repeated	µ agonist	PO, SC, IV, IM, SL, PR	1:2	30-60	30-120	15 8-80	6-8
Morphine	1.0	µ agonist	PO, PR, IV, IM, SC, ID, ED, Topical	1:3 IV 1-2 IM	30-60	60-90	3	4-6
Nalbuphine	1.0	Mixed agonist/ antagonist	SC, IV, IM	1:4- 1:5	15-30	45-60	5	5-6
Tramadol	0.25	µ, O, k agonist + non-opioids	PO, PR, IV, IM, SC	1:4	20-60	30-60	4-6	6
Pethidine	0.125	µ, O, k agonist	PO, SC, IV, IM	1:3	30-60	60-120	2.5	2-4
Codeine	0.1	Prodrug	PO, IM	1:1.5	30	45-60	3	4
Pentazocine	0.06	Mixed agonist/ antagonist	PO, SC, IV, IM	1:4	40-60	60-180	2	2-4

Reproduced from Stannard CF, Booth S. Churchill's Pocketbook of Pain, 2e (Oct 21, 2004) Elsevier Churchill Livingstone, New York, 2004. with permission. ED: Epidural, Oral (PO), Buccal and Sublingual (SL), Rectal (PR), Intravenous (IV), Subcutaneous (SC), Intramuscular (IM), Intradermal (ID), Transmucosal(TM), Topical or Transdermal (TD), Inhalation, Oral/Parenteral Ratio (O:P), Half-Life (T 1/2),

Nonopioid agents such as steroid, nonsteroidal anti-inflammatory drugs, ice, and physical therapy helps with reducing the opioid dose requirement and are effective techniques to prevent long-term sequela from developing. The following topics may provide additional strategies: 

(a) Adjustment or continuation of medications whose sudden cessation may provoke a withdrawal syndrome.

(b) Treatments to reduce preexisting pain and anxiety.

(c) Premedications before surgery as part of a multimodal analgesic pain management program.

Following we summarize most common opioid and non-opioid medications used in pain management:

**Fentanyl:**

Fentanyl, which is a derivate of phenylpiperidine and has a structure close to pethidine is considered hazardous for asthma and cardiac/atherosclerotic patients, because may cause histamine-induced hypotension. Physician should also avoid prescribing fentanyl to patients who have been on MAOIs in previous 14 days. Moreover fentanyl patches compared to IV have longer absorption life after removal (16-20 hours), which is a risk factor for fatal respiratory depression after the pain has subsided. Therefore, many authorities recommend avoiding its use since it offers no advantage for other opioids.^25-27^

**Methadone: **

Another powerful synthetic opioid is methadone, which acts via µ, δ and NMDA (n-methyl-D-aspartate) and its analgesic effect generally last between 6 and 12 hours. When used for acute pain its analgesic effect will last 4-6 hours. ^28^ Although its half-life increases with patient’s age. In the management of severe nociceptive pain, if patients show intolerable reactions, and side effects to morphine, methadone is used as an alternative.^29^ Other situations where the use of methadone is preferred over morphine include when morphine cause allergy, induces pain (paradoxical pain), patient is an opioids abuser, ^30^ and patient is diagnosed with chronic renal failure with neurotoxicity from Morphine-6-glucuronide (M6G) accumulation. The contradictory effects of methadone include respiratory depression, ^31^ severe bronchial asthma, ^32^ paralytic ileus, ^33^ and hypersensitivity (e.g., anaphylaxis). ^34^ Physicians are warned to take precautionary measures when using methadone in combination with antiretroviral drugs in management of HIV/AIDS, ^35^ SSRIs, carbamazepine and phenytoin. ^36^ Furthermore, it is not recommended to use methadone subcutaneously or intraspinally. ^37^

**Morphine:**

Morphine is a powerful µ agonist. Its active metabolite is Morphine-6-glucuronide (M6G), which has analgesic effect and is the first choice in the treatment of severe nociceptive pain. However, once used in high doses, morphine may increase pain. It is suggested that abnormal metabolism of morphine may produce M3G as opposed to M6G.^38^ In such cases the recommendation is to titrate the dose against the effect for each patient and the level of pain, or administer the doses in a 6-8 intervals or longer. ^39^ Particularly when morphine is administered parenterally, fixed dose regimen is not advisable. ^40^

The respiratory depressant effect of morphine in patients with severe head injury or raised intracranial pressure who naturally breathe, may cause an increase in arterial PCO2 causing additional intracranial pressure. Also, for patients with biliary colic, NSAID or pethidine is preferred over morphine, since morphine may increase muscle tone, therefore, pressure, in the biliary tract. Precautionary measures should also be taken with aspirin-sensitive asthma patients, since they are more likely to suffer an allergic reaction to sulphite-contained morphine, ^41^ old patients ^42^ and patients with sleep apnea. ^43^ These may include preparing the apnea resuscitation equipment such as naloxone, oxygen and ambubag. 

**Tramadol: **

Tramadol is a μ-opioid receptor agonist, a metabolite of antidepressant trazodone that inhibits the reuptake of serotonin and norepinephrine.^44,45^ Tramadol is used for the management of moderately severe acute and chronic pain (step 2 analgesic on the WHO ladder).^46^ Structurally Tramadol is related to opioids like drugs such as morphine and codeine, consequently it is considered a habit-forming drug, therefore it should be avoided for patient with history of addiction or at-risk for substance abuse. Its use should be avoided in patients with epilepsy or those susceptible to convulsion.^45,47^ Precautionary measures should be taken when using tramadol with older patient, as well as patients who are diagnosed with kidney or liver diseases.

**Pethidine: **

Pethidine is a synthetic opioid analgesic which has µ agonist properties and anticholinergic effects.^48^ In patients with renal/ hepatic impairment Pethidine is not recommended for chronic pain relief because of metabolite and norpethidine toxicity ^49,50^ causing irritability and nervousness, tremors, myoclonic jerks, twitches and eventually convulsions. ^51^ When Pethidine is used in analgesic doses it produces antitussive effects ^52^ is less constipating than morphine, ^53^ and more emetic.^54^ Pethidine is an irritant solution and should not be used by the subcutaneous route. ^55,56^ Pethidine side-effect in patients who have received monoamine oxidase inhibitors (MAOIs) is controversial. ^57^ However the respiratory depressive, severe hypertensive and dysrhythmias side effects of Pethidine is well reported, as well as its vagolytic activity on the heart rate. ^58-61^

**Codeine:**

Codeine is in WHO Essential Drug List.^62^ It is commonly prescribed post minor general surgery and for management of acute trauma, ^63^ but it is rarely used as a sole analgesic due to its unwanted side effects-primarily nausea and constipation. ^64^ Therefore, the analgesic effect of Codeine has ceiling. ^65^ Codeine works as a prodrug and is converted to morphine in the body. ^66^ Compound analgesics contain both an opioid and a non-opioid. Misuse and dependence of nonprescription codeine analgesics prompt urgent review of chronic pain management. ^67^

**NSAIDs:**

Nonsteroidal Anti-Inflammatory Drugs (NSAIDS) are commonly used for pain management, and analgesic indications (Table 3).^68^ In view of toxicity and side effects, NSAIDs are divided to four categories. Drugs with minimal drug toxicity when prescribed in low doses, e.g. Celecoxib, Rofecoxib, ^69^ Drug with low gut toxicity, e.g. Ibuprofen, Diclofenac, ^70,71^ Drug with intermediate toxicity, e.g. Ketoprofen, Flurbiprofen, ^71^ Drug with very high toxicity, e.g. Azapropazone, Ketorolac. ^72^

**Table 3:** Chemical groups of NSAIDs.

Chemical group	Drugs
Alkanones	Nabumetone
Anthranilic acid	Mefenamic Acid, Floctafenine
Arylpropionic acid	Ibuprofen, Naproxen, Ketoprofen
Enolic acid	Meloxicam, Piroxicam, Tenoxicam
Heteroaryl acetic acid	Diclofenac, Ketorolac
Salicylic acid	Aspirin, Diflunisal
Sulfanilide	Nimesulide
Diarylheterocyclic with sulfa group	Celecoxib, Parecoxib
Diarylheterocyclic with sulfone group	Etoricoxib

Modified from Am J Clin Dermatol 2002; 3(9): 599-607

It must be remembered that the bleeding time will be prolonged until the NSAID has been excreted, except in the case of aspirin, which causes irreversible platelet dysfunction by the acetylation of platelet COX-1. This takes five half-lives of the drug. Adequate time must be left before surgery.^73,74^

**Adjuvant drugs: **

Adjuvant analgesics are primarily used for other conditions than pain but since they have analgesic properties they have been used for painful conditions as well. Adjuvant pain medications can include antidepressants, anti-seizure medications, muscle relaxants, sedatives or anti-anxiety medications, dexmedetomidine, corticosteroid and botulinum toxin.^68,75^ Use of these adjuvant analgesics necessitates comprehensive understanding of their recommended does, side effects, and drug interactions. ^76^

**Routes of administration:**

Since, most of traumatic patients must be nil by mouth (NPO), intravenous route is the most common way to use in traumatic patients. Other routes of administration include: Patient-Controlled Analgesia (PCA), subcutaneous, intramuscular, rectal, transdermal, buccal and sublingual, spinal intrathecal, epidural and inhalation.

The rate of subcutaneous absorption will depend on the local blood flow, therefore, it is not a choice rout in trauma patients since the probability of poor peripheral circulation is higher in these patients. Nonetheless, some drugs are too irritant to be used by the subcutaneous route, for example pethidine, methadone and prochlorperazine.

Rectal route can be used for patient with unconscious, unable to swallow or NPO for any reason, or in cased of nausea or vomiting. Suppositories route in neutropenia may increase the risk of septicemia.^77^ Fentanyl, buprenorphine and EMLA (Eutectic Mixture of Local Anesthetics- Lidocaine/prilocaine) are pain drugs that can be delivered transdermally. However, the reservoir of drug remaining in the skin should always be considered. Transdermal fentanyl has been implicated in death in treatment of acute pain. As the painful stimulus recedes, even if the patch is removed the drug will continue to leach into the systemic circulation and respiratory depression will result.^25,78^

There are a large number of both opioids and non-opioids receptors in the spinal cord which are involved in the modulation of pain signals, some to reduce and some to augment nociception. The term of intraspinal includes both epidural and Intrathecal injection. Intrathecal medication is deposited in the subarachnoid space and epidural drugs are place in the epidural space. The intraspinal route has been used in the management of pain relief in trauma. The most common form of inhaled pain relief is the gas Entonox.

**Regional analgesia:**

Regional analgesia provided through an epidural or brachial catheter should be considered for any trauma patient since this approach had the potential to spare the use of systemic narcotics and facilitate early mobilization. Epidural analgesia has been shown to produce high levels of patient satisfaction and improved pulmonary function after major thoracoabdominal and orthopedic surgery in elective populations^79^ and is likely to be the same for trauma patients. Regional techniques are less practical when the patient has multiple sites of injury or fractures or open wounds. Although epidural placement in anesthetized patients is relatively contraindicated because of the potential for occult spinal cord injury (SCI), the risk-benefit ratio in many trauma patients favors placement during surgery, when general anesthesia facilitates appropriate positioning and patient cooperation.^80^

**Psychological Intervention:**

After an injury patient may have legal, financial, and family-based concerns, without the ability to immediately address them. The availability of counselors—religious, financial, or legal that could help the patient and family with these issues is of enormous benefit. The practitioner can help by communicating to the patient a clear description of the patient's injuries, the probable time required for recovery, and the plan for managing pain throughout the patient's course. The practitioner should refer the patient to counseling services as needed and should be cognizant of possibility of post-traumatic stress disorder (PTSD) in any traumatized patient. Referral to an experienced psychiatrist or psychologist is appropriate if PTSD is hindering the patient's recovery. ^81^

Trauma, because of its unexpected nature, carries with it a strong negative psychological overlay that can have a profound effect on how anatomically based pain is perceived by the brain and on how the patient reacts.^82^ Victims of trauma are frequently frightened and anxious as well as in pain. The importance of explanation and reassurance in this situation, especially prior to examination or investigation, cannot be overemphasized. The need for analgesic medication and the duration of requirement for analgesics will be minimized if a comprehensive emotional support system is available to the patient. 

**3. Re-evaluate:**

Pain assessment must be evaluated and re-evaluated at regular intervals. Pain control must be evaluated and re-evaluated at specific regular intervals.

**4. Pain management after acute phase:**

Neuropathic pain arises when there is direct injury to a major sensory nerve and is common after spinal cord trauma, traumatic amputations, and major crush injuries. Neuropathic pain is characterized by burning, intermittent electrical shocks, and dysesthesia in the affected dermatomal distribution. It is important to identify neuropathic pain because it responds poorly to the analgesics used for nociceptive pain. This diagnosis should be considered whenever pain control is poor or the patient has a rising requirement for medications unexplained by anatomic injuries. 

The first-line therapy for neuropathic pain has been revolutionized by the widespread use of gabapentin, an antiepileptic drug with very strong specificity for this problem.^83^ Gabapentin therapy is typically initiated at a dose of 200 mg three times daily, with daily titration upward to a maximum of 1900-3600 mg/day.^84^

If neuropathic pain persists, selective regional anesthesia or analgesia may be indicated in an effort to “break the cycle” of spinal cord receptor recruitment.^85^

The need for analgesic medication is also influenced by the schedule of physical therapy prescribed for the patient. In general, the more active a patient can be after traumatic injury, the lower the risk of pulmonary complications, venous thrombosis, and decubitus ulcers. Though painful in the short term, the sooner the patient is mobilized, the lower the analgesic requirements in the long term. Early mobilization demonstrates to the patient the “path to recovery” and contributes to an improved emotional state. One of the goals of analgesia, therefore, is provision of adequate medication to facilitate physical therapy without so sedating the patient that participation is impossible.

## Conclusion

The ultimate aim of pain management in trauma is reducing the mortality, morbidity, shortening hospital stay, contributing to early mobilization, and reducing hospital cost, and enhance patient’s satisfaction and quality of life. Traumatic injuries vary in severity from isolated limb fracture to life-threatening multiple bone and soft tissue injuries. Provision of adequate analgesia is a vital component of any system of trauma management that will require: adequate assessment of age-specific pharmacologic pain management; identification of adequate analgesic to relieve moderate to severe pain; cognizance of serious adverse effects of pain medications and weighing that against their benefits, and regularly reassessing the patients and reevaluating their pain management regimen. Patient-centered trauma care will also require having knowledge of barriers to pain management and to discuss them with the patient to identify solution to over come them. 

## Acknowledgments

The authors would like to thank Prof. Wanna Srirojanakul, Department of Anesthesiology and Pain Management, Mahidol University, Bangkok, Thailand, for her helpful suggestions and supports. 

## References

[1] Murray CJ, Lopez AD (1997). Global mortality, disability, and the contribution of risk factors: Global Burden of Disease Study. Lancet.

[2] Peden M, Scurfield R, Sleet D, Mohan D, Hyder A, Jarawan E, et al. World report on road traffic injury prevention. Geneva: World Health Organization, 2004.

[3] Thomas V, Heath M, Rose D, Flory P (1995). Psychological characteristics and the effectiveness of patient-controlled analgesia. Br J Anaesth.

[4] Carroll KC, Atkins PJ, Herold GR, Mlcek CA, Shively M, Clopton P (1999). Pain assessment and management in critically ill postoperative and trauma patients: a multisite study. Am J Crit Care.

[5] Hofman K, Primack A, Keusch G, Hrynkow S (2005). Addressing the growing burden of trauma and Injury in Low- and middle-income countries. Am J Public Health.

[6] Mock CN, Jurkovich GJ, nii-Amon-Kotei D, Arreola-Risa C, Maier RV (1998). Trauma mortality patterns in three nations at different economic levels: implications for global trauma system development. J Trauma.

[7] Cohen SP, Christo PJ, Moroz L (2004). Pain management in trauma patients. Am J Phys Med Rehabil.

[8] Gausche-Hill M, Brown KM, Oliver ZJ, Sasson C, Dayan PS, Eschmann NM (2014). An evidence-based guideline for prehospital analgesia in trauma. Prehosp Emerg Care.

[9] Hedderich R, Ness TJ (1999). Analgesia for trauma and burns. Crit Care Clin.

[10] Aisuodionoe-Shadrach O, Olapade-Olaopa EO, Soyannwo OA (2006). Preoperative analgesia in emergency surgical care in Ibadan. Trop Doct.

[11] Woolf CJ, Salter MW (2000). Neuronal plasticity: increasing the gain in pain. Science.

[12] Cohen SP, Christo PJ, Moroz L (2004). Pain management in trauma patients. Am J Phys Med Rehabil.

[13] Malchow RJ, Black IH (2008). The evolution of pain management in the critically ill trauma patient: Emerging concepts from the global war on terrorism. Crit Care Med.

[14] Anand KJ, Hickey PR (1992). Halothane–morphine compared with high-dose sufentanil for anesthesia and postoperative analgesia in neonatal cardiac surgery. N Engl J Med.

[15] Yeager MP, Glass DD, Neff RK, Brinck-Johnsen T (1987). Epidural anesthesia and analgesia in high-risk surgical patients. Anesthesiology.

[16] Whipple JK, Lewis KS, Quebbeman EJ, Wolff M, Gottlieb MS, Medicus-Bringa M (1995). Analysis of pain management in critically ill patients. Pharmacotherapy.

[17] Nair V, Kaduskar M, Bhaskaran P, Bhaumik S, Lee H (2011). Preserving narratives in electronic health records, Bioinformatics and biomedicine (BIBM), 2011 IEEE international conference on: 2011. Atlanta: IEEE.

[18] Chapman K, Rush K (2003). Patient and family satisfaction with cancer-related information: a review of the literature. Can Oncol Nurs J.

[19] Worle Health Organization. WHO’s Pain Relief Ladder. http://www.who.int/cancer/palliative/painladder/en/ , accessed 19 January 2005.

[20] Roden A, Sturman E (2009). Assessment and management of patients with wound-related pain. Nurs Stand.

[21] A World Union of Wound Healing Societies’ Initiative. Principles of best practice: minimising pain at wound dressing-related procedures. A consensus document.Parise:WUWHS, 2007.

[22] Reuben SS, Ekman EF (2007). The effect of initiating a preventive multimodal analgesic regimen on long-term patient outcomes for outpatient anterior cruciate ligament reconstruction surgery. Anesth Analg.

[23] Reuben SS, Buvanendran A (2007). Preventing the development of chronic pain after orthopaedic surgery with preventive multimodal analgesic techniques. J Bone Joint Surg Am.

[24] Dowden SJ. Pharmacology of analgesic drugs. In: Twycross A, Dowden SJ, Bruce E (eds).Managing Pain in Children: a clinical guide. Oxford (UK): Wiley-Blackwell, 2009:39-66.

[25] Grissinger M (2010). Inappropriate prescribing of fentanyl patches is still causing alarming safety problems. P T.

[26] Weaver JM (2014). Multiple risks for patients using the transdermal fentanyl patch. Anesth Prog.

[27] US.Food and Drug Adminstration.Information for Healthcare Professionals: Fentanyl Transdermal System (marketed as Duragesic and generics. http://www.fda.gov/Drugs/DrugSafety/PostmarketDrugSafetyInformationforPatientsandProviders/DrugSafetyInformationforHeathcareProfessionals/ucm084307.htm.),accessed 12 December 2007.

[28] Lynch ME (2005). A review of the use of methadone for the treatment of chronic noncancer pain. Pain Res Manag.

[29] Morley JS, Watt JW, Wells JC, Miles JB, Finnegan MJ, Leng G (1993). Methadone in pain uncontrolled by morphine. Lancet.

[30] Alford DP, Compton P, Samet JH (2006). Acute pain management for patients receiving maintenance methadone or buprenorphine therapy. Ann Intern Med.

[31] Tarumi Y, Pereira J, Watanabe S (2002). Methadone and fluconazole: respiratory depression by drug interaction. J Pain Symptom Manage.

[32] Rosenow EC, Myers JL, Swensen SJ, Pisani RJ (1992). Drug-induced pulmonary disease. An update. Chest.

[33] Kurz A, Sessler DI (2003). Opioid-induced bowel dysfunction: pathophysiology and potential new therapies. Drugs.

[34] Angst MS, Clark JD (2006). Opioid-induced hyperalgesia:a qualitative systematic review. Anesthesiology.

[35] Bruce RD, Altice FL, Gourevitch MN, Friedland GH (2006). Pharmacokinetic drug interactions between opioid agonist therapy and antiretroviral medications: implications and management for clinical practice. J Acquir Immune Defic Syndr.

[36] Mercadante S, Casuccio A, Fulfaro F, Groff L, Boffi R, Villari P (2001). Switching from morphine to methadone to improve analgesia and tolerability in cancer patients: a prospective study. J Clin Oncol.

[37] Hassenbusch SJ, Portenoy RK, Cousins M, Buchser E, Deer TR, Du Pen SL (2004). Polyanalgesic Consensus Conference 2003: an update on the management of pain by intraspinal drug delivery—report of an expert panel. J Pain Symptom Manage.

[38] Andersen G, Christrup L, Sjøgren P (2003). Relationships among morphine metabolism, pain and side effects during long-term treatment: an update. J Pain Symptom Manage.

[39] Hanks GW, Forbes K (1997). Opioid responsiveness. Acta Anaesthesiol Scand.

[40] McNicol E, Horowicz-Mehler N, Fisk RA, Bennett K, Gialeli-Goudas M, Chew PW (2003). Management of opioid side effects in cancer-related and chronic noncancer pain: a systematic review. J Pain.

[41] Golembiewski JA (2002). Allergic reactions to drugs: implications for perioperative care. J Perianesth Nurs.

[42] Daykin A, Bowen DJ, Saunders DA, Norman J (1986). Respiratory depression after morphine in the elderly. A comparison with younger subjects. Anaesthesia.

[43] Lam KK, Kunder S, Wong J, Doufas AG, Chung F (2016). Obstructive sleep apnea, pain, and opioids: is the riddle solved?. Curr Opin Anaesthesiol.

[44] Drug.com. Know more. Be sure. https://www.drugs.com/tramadol.html,accessed 10 Nov 2015.

[45] McCarberg B (2007). Tramadol extended-release in the management of chronic pain. Ther Clin Risk Manag.

[46] Akcali GE, Iskender A, Demiraran Y, Kayikci A, Yalcin GS, Cam K (2010). Randomized comparison of efficacy of paracetamol, lornoxicam, and tramadol representing three different groups of analgesics for pain control in extracorporeal shockwave lithotripsy. J Endourol.

[47] Boostani R, Derakhshan S (2012). Tramadol induced seizure: A 3-year study. Caspian J Intern Med.

[48] Sear JW (1998). Recent advances and developments in the clinical use of i.v. opioids during the peroperative period. Br J Anaesth.

[49] Stone PA, Macintyre PE, Jarvis DA (1993). Norpethidine toxicity and patient controlled analgesia. Br J Anaesth.

[50] O'Connor A, Schug SA, Cardwell H (2000). A comparison of the efficacy and safety of morphine and pethidine as analgesia for suspected renal colic in the emergency setting. J Accid Emerg Med.

[51] Danziger LH, Martin SJ, Blum RA (1994). Central nervous system toxicity associated with meperidine use in hepatic disease. Pharmacotherapy.

[52] Eddy NB, Friebel H, Hahn KJ, Halbach H (1969). Codeine and its alternates for pain and cough relief: 4. Potential alternates for cough relief. Bull World Health Organ.

[53] McQuay H (1999). Opioids in pain management. Lancet.

[54] Lehmann KA (1997). [Tramadol in acute pain].. Drugs.

[55] Argent DE, Dinnick OP (1954). Pethidine phlebitis. Br J Anaesth.

[56] Oldroyd GJ, Tham EJ, Power I (1994). An investigation of the local anaesthetic effects of pethidine in volunteers. Anaesthesia.

[57] Stack CG, Rogers P, Linter SP (1988). Monoamine oxidase inhibitors and anaesthesia. A review. Br J Anaesth.

[58] Tarkkila P, Tuominen M, Lindgren L (1998). Comparison of respiratory effects of tramadol and pethidine. Eur J Anaesthesiol.

[59] Lawrence CA (1978). Pethidine-induced hypertension in phaeochromocytoma. Br Med J.

[60] Pant KK, Verma VK, Mishra N, Singh N, Sinha JN, Bhargava KP (1983). Effects of morphine and pethidine on coronary vascular resistance, blood pressure, and myocardial infarction-induced cardiac arrhythmias. Jpn Heart J.

[61] Owitz S, Pratilas V, Pratila MG, Dimich I (1979). Anaesthetic considerations in the prolonged Q-T interval (LQTS): a case report. Can Anaesth Soc J.

[62] Kasim NA, Whitehouse M, Ramachandran C, Bermejo M, Lennernäs H, Hussain AS (2004). Molecular properties of WHO essential drugs and provisional biopharmaceutical classification. Mol Pharm.

[63] Vira A. Codeine and codeine compounds. The Essence of Analgesia and Analgesics . Cambridge: Cambridge University Press, 2010:98.

[64] Gonenne J, Camilleri M, Ferber I, Burton D, Baxter K, Keyashian K (2005). Effect of alvimopan and codeine on gastrointestinal transit: a randomized controlled study. Clin Gastroenterol Hepatol.

[65] Goldsack C, Scuplak SM, Smith M (1996). A double-blind comparison of codeine and morphine for postoperative analgesia following intracranial surgery. Anaesthesia.

[66] Eckhardt K, Li S, Ammon S, Schänzle G, Mikus G, Eichelbaum M (1998). Same incidence of adverse drug events after codeine administration irrespective of the genetically determined differences in morphine formation. Pain.

[67] Roussin A, Bouyssi A, Pouché L, Pourcel L, Lapeyre-Mestre M (2013). Misuse and dependence on non-prescription codeine analgesics or sedative H1 antihistamines by adults: a cross-sectional investigation in France. PloS One.

[68] Portenoy RK (2000). Current pharmacotherapy of chronic pain. J Pain Symptom Manage.

[69] Ray WA, Stein CM, Daugherty JR, Hall K, Arbogast PG, Griffin MR (2002). COX-2 selective non-steroidal anti-inflammatory drugs and risk of serious coronary heart disease. Lancet.

[70] García Rodríguez LA (1998). Variability in risk of gastrointestinal complications with different nonsteroidal anti-inflammatory drugs. Am J Med.

[71] Trewin VF, Lawrence CJ, Rae SA, Veitch GB (1994). Development and use of a gastropathy index for ranking the safety of non-steroidal anti-inflammatory drugs in the elderly. J Clin Pharm Ther.

[72] Macario A, Lipman AG (2001). Ketorolac in the era of cyclo-oxygenase-2 selective nonsteroidal anti-inflammatory drugs: a systematic review of efficacy, side effects, and regulatory issues. Pain Med.

[73] Walder B, Schafer M, Henzi I, Tramer MR (2001). Efficacy and safety of patient-controlled opioid analgesia for acute postoperative pain. A quantitative systematic review. Acta Anaesthesiol Scand.

[74] Schafer AI (1999). Effects of nonsteroidal anti-inflammatory therapy on platelets. Am J Med.

[75] Grosu I, Lavand'homme P (2010). Use of dexmedetomidine for pain control. F1000 Med Rep.

[76] Lussier D, Huskey AG, Portenoy RK (2004). Adjuvant analgesics in cancer pain management. Oncologist.

[77] Adelberg DE, Bishop MR (2009). Emergencies related to cancer chemotherapy and hematopoietic stem cell transplantation. Emerg Med Clin North Am.

[78] U.S.Food and Drug Administration.Information for Healthcare Professionals: Fentanyl Transdermal System (marketed as Duragesic and generics. http://www.fda.gov/Drugs/DrugSafety/PostmarketDrugSafetyInformationforPatientsandProviders/DrugSafetyInformationforHeathcareProfessionals/ucm084307.htm), accessed 15 July 2005.

[79] Holte K, Kehlet H (2002). Epidural anaesthesia and analgesia–effects on surgical stress responses and implications for postoperative nutrition. Clin Nutr.

[80] Ballantyne JC, Carr DB, Chalmers TC, Dear KB, Angelillo IF, Mosteller F (1993). Postoperative patient-controlled analgesia: meta-analyses of initial randomized control trials. J Clin Anesth.

[81] Breslau N (2009). The epidemiology of trauma, PTSD, and other posttrauma disorders. Trauma Violence Abuse.

[82] Beecher HK (1956). Relationship of significance of wound to pain experienced. J Am Med Assoc.

[83] Backonja M, Glanzman RL (2003). Gabapentin dosing for neuropathic pain: evidence from randomized, placebo-controlled clinical trials. Clin Ther.

[84] O'Connor AB, Dworkin RH (2009). Treatment of neuropathic pain: an overview of recent guidelines. Am J Med.

[85] Cossins L, Okell RW, Cameron H, Simpson B, Poole HM, Goebel A (2013). Treatment of complex regional pain syndrome in adults: a systematic review of randomized controlled trials published from June 2000 to February 2012. Eur J Pain.

